# Unveiling the biochemical signature of tumor-induced osteomalacia: implications for distinguishing it from primary osteoporosis

**DOI:** 10.3389/fendo.2026.1899331

**Published:** 2026-07-24

**Authors:** Yun Chai, Xin Wang, Sen Li, Zhiwang Wei, Qiusong Chen, Baoping Wang, Qing He, Ming Liu, Wenli Feng

**Affiliations:** 1 Department of Endocrinology and Metabolism, Tianjin Medical University General Hospital, Tianjin, China; 2 Department of General Internal Medicine, The Affiliated Hospital of Nankai University, Tianjin, China; 3 Department of General Surgery,Tianjin Medical University General Hospital, Tianjin, China; 4 Department of PET-CT Diagnostic,Tianjin Medical University General Hospital, Tianjin, China

**Keywords:** diagnostic delay, differential diagnosis, fibroblast growth factor 23, primary osteoporosis, tumor-induced osteomalacia

## Abstract

**Purpose:**

Tumor-induced osteomalacia (TIO) is a rare paraneoplastic syndrome caused by FGF23-secreting tumors, resulting in renal phosphate wasting and hypophosphatemic osteomalacia. Low bone mineral density (BMD) in TIO frequently leads to misdiagnosis as the more prevalent primary osteoporosis (POP) and subsequent diagnostic delay. This study characterizes the distinct biochemical profile of TIO and proposes a stepwise diagnostic approach to differentiate it from POP for timely clinical identification and appropriate management.

**Methods:**

We conducted a retrospective comparative analysis of 11 TIO patients and 12 POP patients managed at our institution over a 14-year period. Detailed clinical data were collected and compared between groups, including history, physical examination findings, laboratory results, imaging studies, and pathological outcomes.

**Results:**

Comparative analysis revealed that, compared with POP patients, TIO patients had significantly lower serum calcium (2.19 ± 0.1 vs.2.3 ± 0.1 mmol/L, *p* < 0.05), serum phosphorus [0.46 (0.42, 0.6) vs. 1.15 (1.13, 1.23), *p* < 0.001], and the tubular maximum reabsorption of phosphate per glomerular filtration rate (TmP/GFR) (0.41 ± 0.2 vs.1.05 ± 0.15 mmol/L, *p* < 0.001). In contrast, ALP was significantly higher in the TIO cohort [317 (177.5, 344) vs. 79.5 (67.75, 86.75) U/L, *p* < 0.001]. No significant intergroup differences were observed in PTH levels or BMD.

**Conclusions:**

We recommend serum phosphate, alkaline phosphatase, and TmP/GFR tests for symptomatic patients (e.g., bone pain, muscle weakness). Confirmed hypophosphatemia warrants FGF23 testing after excluding other causes of elevation. Subsequent bone X-ray and BMD guides the need for molecular imaging. The diagnostic gold standard comprises: (1) histopathological confirmation of the causative tumor, (2) rapid postoperative serum phosphate normalization, and (3) significant clinical improvement.

## 1 Introduction

Disorders of bone mineral metabolism serve as a frequent pathological basis for increased skeletal fragility and susceptibility to fractures. Primary osteoporosis (POP), a systemic skeletal disorder defined by diminished bone mass and impaired microarchitecture, represents a major global public health challenge owing to its elevated prevalence—notably among aging and postmenopausal female populations—and the considerable burden of resulting fractures. The diagnostic and therapeutic framework for POP is relatively well-defined, centering on bone mineral density assessment, fracture risk evaluation, and interpretation alongside standard biochemical markers (e.g., serum calcium, phosphorus, alkaline phosphatase).

In clinical practice, however, a distinct disease entity—tumor-induced osteomalacia (TIO)—may present with features resembling POP, including bone pain, loss of height, functional limitation, and low-trauma or atraumatic fractures, yet it arises from a fundamentally divergent pathophysiology. TIO is a rare paraneoplastic syndrome most commonly driven by fibroblast growth factor 23 (FGF23)-secreting mesenchymal tumors termed phosphaturic mesenchymal tumors (PMTs), which are typically benign, small, and anatomically occult (1, 2). Excessive FGF23 production impairs renal phosphate reabsorption, resulting in hypophosphatemia, and suppresses active vitamin D synthesis. These disturbances subsequently compromise bone mineralization, leading to osteomalacia as the hallmark skeletal manifestation (3–5). The underlying metabolic disturbance in TIO is fundamentally distinct from the mechanisms that drive bone mass reduction and microarchitectural deterioration in POP.

Early recognition and precise diagnosis of TIO are of critical clinical importance. First, timely localization and surgical excision of the causative tumor represent the sole curative intervention for TIO, often resulting in marked symptomatic relief (e.g., relief of bone pain) and recovery of skeletal mineralization postoperatively. However, TIO is commonly misdiagnosed as refractory osteoporosis or osteoarthritis. This diagnostic error leads to prolonged administration of inappropriate and ineffective anti-osteoporotic agents (such as bisphosphonates or denosumab), which not only delays tumor detection and appropriate management but also contributes to progressive deterioration of bone mineralization, muscle weakness, functional decline, and may eventually cause irreversible skeletal deformities and severe pathological fractures (3, 6).

Regrettably, TIO is often subject to delayed or missed diagnosis in clinical practice, attributable to its low incidence, the frequently occult nature of the causative tumor, and overlapping symptoms with POP (7). Standard bone mineral density measurement offers limited utility in distinguishing between these two conditions (8). Consequently, a critical strategy for improving early TIO recognition lies in leveraging readily accessible clinical data from routine hospital evaluations, particularly distinctive patterns within biochemical laboratory profiles.

This retrospective case-control study systematically compares key hospitalization-based laboratory data between TIO and POP patients, focusing on patterns of core biomarkers for calcium-phosphate homeostasis, bone turnover, and renal function (e.g., serum and urine phosphate/calcium, PTH, ALP, FGF23, 25(OH)D, and tubular reabsorption of phosphate). We aim to define a TIO-specific laboratory profile to help distinguish it from POP in patients with non-specific bone symptoms or unexplained low BMD. Our ultimate goal is to reduce diagnostic delays and improve TIO prognosis.

## 2 Materials and methods

### 2.1 Patients and data collection

A retrospective analysis was performed on 11 consecutive TIO patients and 12 POP patients admitted to the Endocrinology and Metabolism Department of Tianjin Medical University General Hospital from September 2011 to September 2025. Comprehensive data extraction from medical records encompassed detailed demographics, clinical history, physical and laboratory examinations, imaging findings, and pathological diagnoses when available. Specifically, we retrieved information on pre- and post-treatment biochemical parameters, prior misdiagnoses, tumor localization and detection modalities, procedures and outcomes of any lesion biopsies, chosen therapeutic interventions, histological characteristics of resected tumors, treatment responses, and persistence of symptoms.

#### 2.1.1 Inclusion criteria

(1) age 18 years or older; (2) biochemical confirmation of TIO, defined as serum phosphorus below the lower limit of normal (<0.8 mmol/L), an elevated serum fibroblast growth factor 23 (FGF23) concentration (>95.4 pg/mL), and a reduced tubular maximum phosphate reabsorption rate per glomerular filtration rate (TmP/GFR) (<0.8 mmol/L); and (3) radiographic localization of the responsible tumor using anatomic imaging (e.g., CT, MRI) and functional/metabolic imaging (e.g., PET-CT).

#### 2.1.2 Exclusion criteria

##### 2.1.2.1 TIO cohort

We excluded individuals with any history of malignancy, with the exception of the tumor identified as the cause of TIO in each enrolled patient [e.g., phosphaturic mesenchymal tumors (PMTs)]. Additional exclusions comprised subjects with renal dysfunction or nephrocalcinosis-related renal failure.

##### 2.1.2.2 POP cohort

To establish a primary osteoporosis cohort, we excluded patients with known causes of secondary osteoporosis, including hematologic malignancies (e.g., multiple myeloma), skeletal metastases, autoimmune and endocrine disorders (e.g., hyperparathyroidism, hyperthyroidism, Cushing’s syndrome, acromegaly), and other identifiable secondary etiologies.

### 2.2 Biochemical parameters

#### 2.2.1 Demographic and clinical characteristics

Data on demographics (age, sex), disease history (duration), clinical presentation (including symptoms such as bone pain location, history of fractures, height loss, and functional impairment; and signs such as skeletal deformities), treatment details, and follow-up information were systematically collected.

#### 2.2.2 Laboratory investigations

We retrieved blood and urine test results from the electronic medical record system of Tianjin Medical University General Hospital. The laboratory parameters collected included: serum phosphorus, serum calcium, creatinine (CREA), alkaline phosphatase (ALP), parathyroid hormone (PTH), 24-hour urinary phosphate, 24-hour urinary calcium, total 25-hydroxyvitamin D [25(OH)D], and three bone turnover markers: osteocalcin (OC), total procollagen type I N-terminal propeptide (PINP), and β-C-terminal telopeptide of type I collagen (β-CTX). The reference ranges for each parameter are as follows:

Serum phosphorus reference range: 0.80-1.45mmol/L
Serum calcium reference range: 2.15-2.55mmol/L.
Creatinine (enzymatic assay) reference range: 41-81umol/L.
Alkaline phosphatase (ALP) reference range: 50–135 U/L
Parathyroid hormone (PTH) reference range: 1.10–7.30 pmol/L
24-hour urinary phosphate was calculated as urinary phosphate concentration (mmol/L) multiplied by 24-hour urine volume (L). Reference range: 23–48 mmol/24h.
24-hour urinary calcium was calculated as urinary calcium concentration (mmol/L) multiplied by 24-hour urine volume (L). Reference range: 2.5-7.5 mmol/24h.
25-Hydroxyvitamin D [25(OH)D] levels were categorized as deficient (<20 ng/mL), insufficient (20–30 ng/mL), or sufficient (>30 ng/mL).
Osteocalcin (OC) reference range: 11.00–48.00 ng/mL.
β-C-terminal telopeptide of type I collagen (β-CTX) reference range: 0.30–0.57 ng/mL (premenopausal); 0.55–1.01 ng/mL (postmenopausal).
Procollagen type I N-terminal propeptide (PINP) reference range: 19.00–84.00 ng/mL.

#### 2.2.3 TRP and TmP/GFR

Tubular reabsorption of phosphate (TRP) was calculated as per Walton and Bijvoet (9): TRP = 1 – [(Up × Scr)/(Sp × Ucr)], where Up and Ucr represent fasting urinary phosphate and creatinine concentrations, respectively, and Sp and Scr represent the same-day serum phosphate and creatinine concentrations. From TRP, the tubular maximum for phosphate reabsorption normalized to glomerular filtration rate (TmP/GFR) was computed using established conditional equations: For TRP ≤ 0.86, TmP/GFR = TRP × Sp; for TRP > 0.86, TmP/GFR = (0.3 × TRP)/[1 – (0.8 × TRP)] × Sp.

#### 2.2.4 Serum fibroblast growth factor 23

The concentration of intact fibroblast growth factor 23 (iFGF23) was measured in EDTA plasma samples employing a three-step sandwich chemiluminescent immunoassay (CLIA). This assay is specific for the biologically active, full-length iFGF23 polypeptide, thereby quantifying the circulating level of the phosphaturic hormone secreted by osteocytes, and does not cross-react with carboxyl-terminal (cFGF23) or amino-terminal (nFGF23) fragments.

Assays for study participants were conducted at the laboratory of Kingmed Diagnostics in Boao, Hainan, using the manufacturer’s (DiaSorin) CLIA kit on a LIAISON XL automated analyzer. The established reference upper limit of normal, derived from internal validation against healthy controls and corroborated by literatures (10), was 95.4 pg/mL. Values exceeding this threshold were considered indicative of pathological FGF23 elevation.

#### 2.2.5 Detection of the tumor

For tumor localization, a functional imaging-based approach was utilized. With advancements in imaging technology, two primary modalities are currently employed: 68Ga-labeled somatostatin analogs PET/CT (68Ga-DOTATATE PET/CT) and 18F-fluorodeoxyglucose PET/CT (18F-FDG PET/CT). Following the identification of any suspicious uptake, confirmatory anatomical imaging (such as X-ray, CT, or MRI) was obtained to better characterize the tumor and its spatial relationship to adjacent anatomy.

#### 2.2.6 Histopathology

In this study, 9 TIO patients underwent surgical treatment, and the resected lesions were submitted for pathological examination. According to the latest WHO classification of tumors (11), the histological diagnosis of PMT is defined as follows: the essential criterion is the presence of “a usually bland spindle cell tumor with matrix deposition, grungy calcification, and/or a rich vascular network,” supported by clinical evidence of hypophosphatemia and/or osteomalacia.

#### 2.2.7 Statistical analysis

A minimal amount of missing data in laboratory and imaging parameters (e.g., TmP/GFR in some patients from both cohorts) was assumed to be missing at random and was addressed using multiple imputation. Normality tests were performed for continuous variables. Normally distributed data are presented as mean ± standard deviation (SD). Non-normally distributed data are expressed as median (interquartile range, IQR). Categorical variables are presented as frequency (percentage) [n (%)]. For between-group comparisons, independent samples t-tests were used for normally distributed continuous data. The Mann-Whitney U test was applied for non-normally distributed continuous data. For categorical variables, the Chi-square test or Fisher’s exact test (if expected cell counts were <5) was used. All statistical analyses were performed using SPSS (version 27.0) and R (version 4.3.3).

### 2.3 Ethical considerations

The study was conducted in accordance with the principles of the Declaration of Helsinki. Written informed consent was obtained from all participants.

## 3 Results

### 3.1 General characteristics of TIO patients

This retrospective analysis included consecutive TIO and primary osteoporosis (POP) patients managed from March 2011 to September 2025. Key characteristics of the TIO cohort are detailed in **Table 1**.

**Table 1:** Patients’ characteristics and tumor localization.

Subjects	Gender	Age at symptoms onset	Age at diagnosis	Tumor location
1	M	50	52	Soft tissue (subcutaneous abdomen)
2	F	53	56	Left femoral shaft
3	F	45	48	Right mandible
4	F	39	40	Soft tissue (subcutaneous tissue on the left side of the buttocks)
5	F	43	45	Mandible
6	F	61	66	Near the proximal end of the right femur
7	M	40	43	Right mandible
8	M	50	52	Left nasal-cranial base
9	M	74	76	Cervical spine, C4 spinous process
10	M	5	23	Near the proximal end of the left femur
11	M	58	62	Deep surface of the patella in the right knee joint

**Figure 1:**
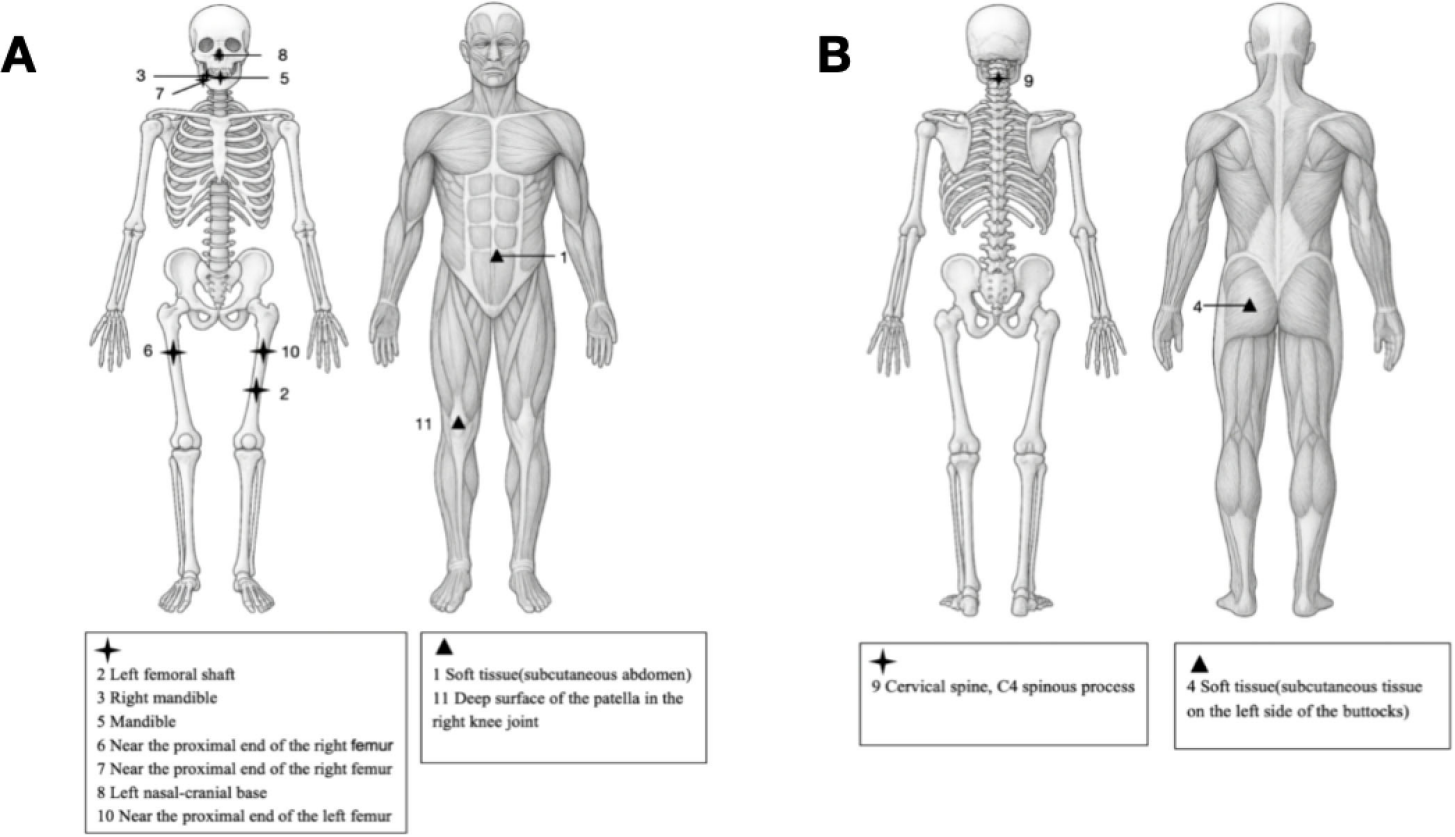
Anatomic distribution map of tumor localizations. **(A)** Anterior and **(B)** posterior views showing the distribution of causative tumors.

The TIO patients were diagnosed at a mean (± SD) age of 51 years (± 14 years), with symptoms first appearing at 47 years (± 17 years), resulting in a median diagnostic delay of 36 months (24–48 months). All patients were previously misdiagnosed once or many times, most commonly with osteoporosis (3 cases) or lumbar disc herniation (3 cases). Other prior diagnoses included hematologic disorders (1 case), hypophosphatemic rickets (1 case), and secondary hyperparathyroidism (1 case); 2 patients remained undiagnosed. Clinical features comprised bone pain (100%), muscle weakness/fatigue (45%), gait impairment (45%), height loss (9%), and pathological fractures (55%). BMD assessment indicated reduced bone mass in 7 patients (4 osteoporotic, 3 osteopenic). A further 4 patients had documented BMD results from external institutions consistent with osteoporosis/osteopenia. To mitigate measurement variability attributable to differing equipment and technical protocols, these latter results were excluded from the formal analysis. Fractures preceded TIO diagnosis in 55% of cases. Tumor locations are illustrated in **Figure 1**.

### 3.2 Biochemical profile and comparative analysis between TIO and primary osteoporosis patients

**Table 2A** summarizes the biochemical profile, including serum calcium, phosphorus, alkaline phosphatase (ALP), parathyroid hormone (PTH), 25-hydroxyvitamin D [25(OH)D], 24-hour urinary calcium and phosphate, TmP/GFR and FGF23, for the 11 TIO patients. The analogous profile (excluding FGF23) for the 12 POP patients is provided in **Table 2B**.

**Table 2A:** Biochemical profile of TIO patients.

TIO	SCa	SP	ALP	PTH	25 (OH)D	24hUCa	24hUP	TmP/ GFR	FGF23
	(mmol/L)	(mmol/L)	(U/L)	(pmol/L)	(nmol/L)	(mmol/24h)	(mmol/24h)	(mmol/L)	(pg/ml)
1	2.14	0.39	425	7.3	19.14	7.64	20.48	–	–
2	2.07	0.43	224	10.6	55.41	1.49	35.38	0.24	–
3	1.99	0.46	221	17.2	20.675	0.72	6.58	0.37	–
4	2.29	0.62	332	10.6	19.66	5.85	18.5	0.57	–
5	2.29	0.59	133	4.98	38.7	2.1	7.1	0.68	–
6	2.22	0.4	134	5.54	36.55	6.26	53.51	0.09	324.6
7	2.09	0.56	317	15.1	24.26	3.6	23.68	0.43	317
8	2.26	0.42	345	4.23	28.55	1.24	18.26	0.27	324
9	2.27	0.41	355	3.95	27.975	2.01	33.48	0.38	476.6
10	2.27	0.72	132	7.76	68.47	3.71	24.75	0.62	–
11	2.17	0.71	343	8.32	77.36	0.37	20.75	0.65	–

**Table 2B:** Biochemical profile of patients with primary osteoporosis.

POP patient	SCa	SP	ALP	PTH	25 (OH)D	24hUCa	24hUP	TmP/GFR
	(mmol/L)	(mmol/L)	(U/L)	(pmol/L)	(nmol/L)	(mmol/24h)	(mmol/24h)	(mmol/L)
1	2.33	1.14	68	5.55	20.7	4.31	25.06	0.97
2	2.25	1.29	153	15.7	14.77	4.06	13.48	0.92
3	2.41	1.18	156	5.54	18.55	6.8	28.83	1.13
4	2.36	1.12	85	1.87	68.45	3.84	20.44	0.99
5	2.26	1.15	77	2.73	46.34	5.1	26.45	1.05
6	2.23	1.24	67	5.88	47.5	4.55	21.49	1.28
7	2.41	1.3	69	3.5	19.11	1.89	16.1	–
8	2.36	1.15	65	2.23	32.73	10.3	41.04	–
9	2.14	1.13	84	36.7	45.39	7.47	16.77	–
10	2.32	1.22	82	3.54	51.61	1.75	22.45	–
11	2.13	1	59	4.28	26.25	6.37	37.77	0.88
12	2.37	1.12	92	4.61	36.35	6	12.98	1.188

Comparative analysis (**Table 3**) revealed that TIO patients had significantly lower serum calcium, phosphorus, and TmP/GFR levels relative to POP patients. In contrast, ALP activity was significantly higher in the TIO cohort. No statistically significant intergroup differences were detected for PTH, 25(OH)D, 24-hour urinary calcium, or 24-hour urinary phosphate.

**Table 3:** Comparative analysis of biochemical profiles between TIO and primary osteoporosis patients.

Biochemical profiles	TIO	POP	p
SCa (mmol/L)	2.19 ± 0.1	2.3 ± 0.1	0.015
SP (mmol/L)	0.46 (0.42, 0.6)	1.15 (1.13, 1.23)	< 0.001
ALP (U/L)	317 (177.5, 344)	79.5 (67.75, 86.75)	< 0.001
PTH (pmol/L)	7.76 (5.26, 10.6)	4.44 (3.31, 5.63)	0.074
25(OH)D (nmol/L)	28.55 (22.47, 47.06)	34.54 (20.3, 46.63)	0.74
24hUCa (mmol/24h)	3.18 ± 2.45	5.2 ± 2.39	0.059
24hUP (mmol/24h)	23.86 ± 13.29	23.57 ± 8.93	0.952
TmP/GFR (mmol/L)	0.41 ± 0.2	1.05 ± 0.15	< 0.001

**Figure 2:**
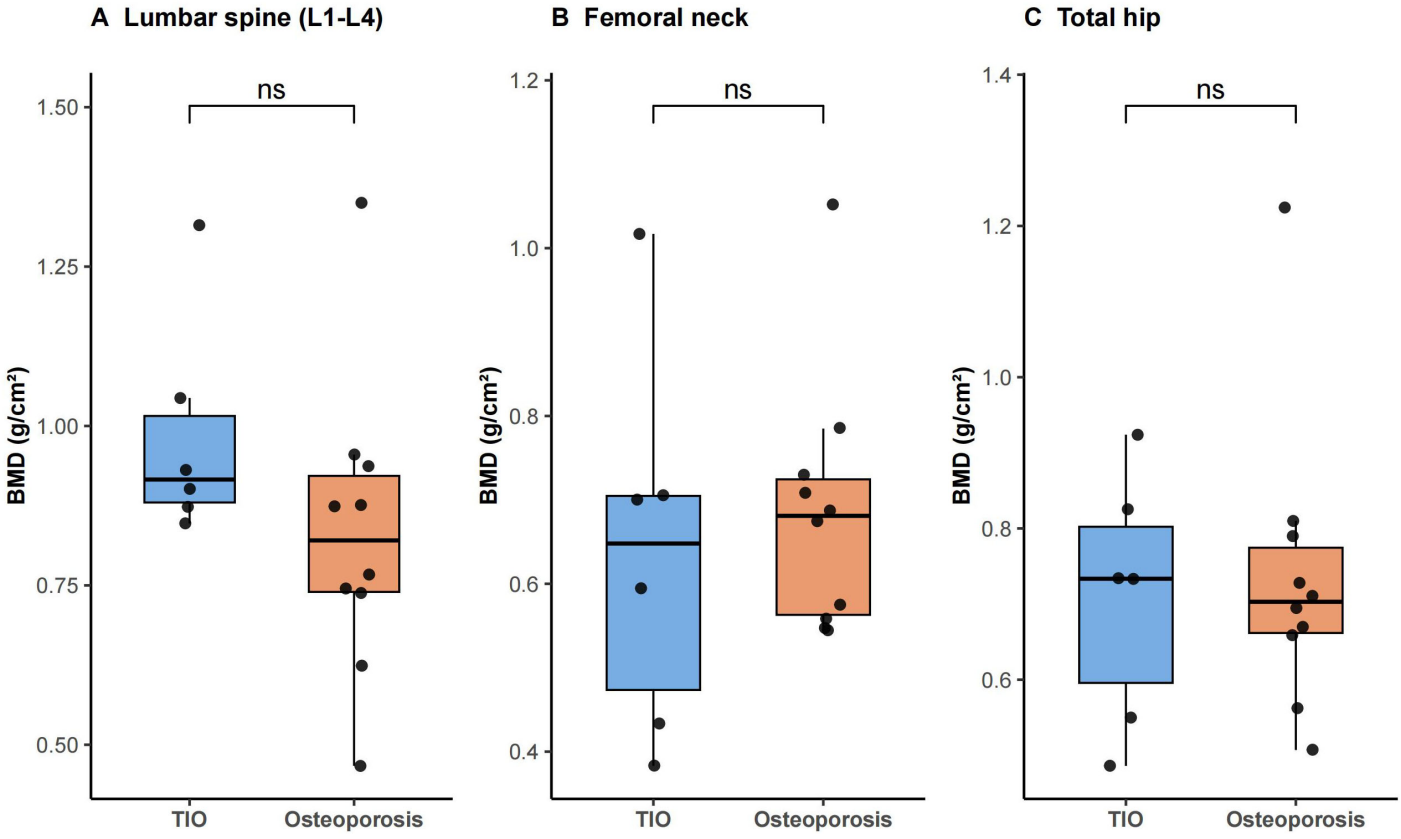
Comparison of bone mineral density(BMD) between patients with TIO and primary osteoporosis. **(A)** Lumbar spine (L1-L4); **(B)** Femoral neck; **(C)** Total hip.

BMD values did not differ significantly between the TIO and POP groups (**Figure 2**).

### 3.3 Therapy and outcome

#### Pre- versus post-operative parameter changes

**Figure 3:**
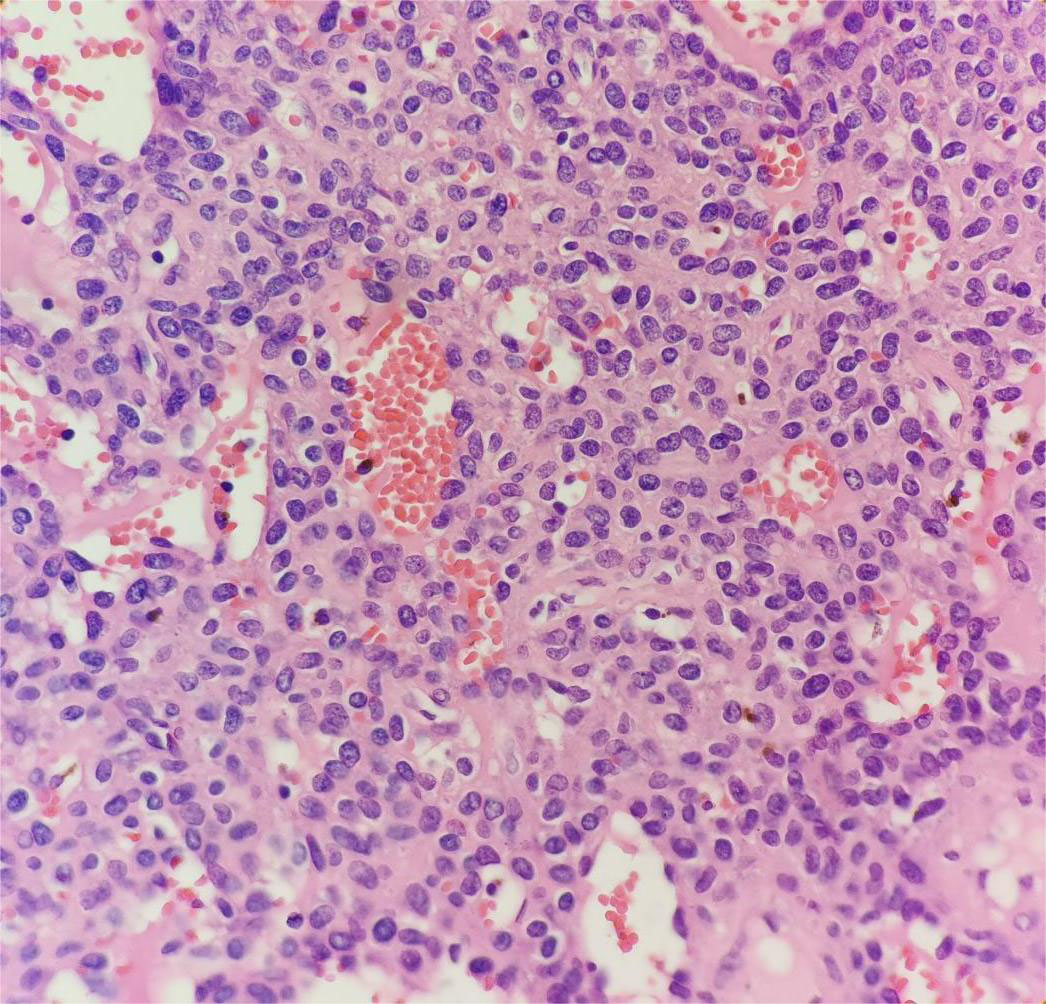
E & E 20×. Phosphaturic mesenchymal tumor with ovoid to short spindle-shaped cells, accompanied by focal stromal sclerosis and occasional calcific deposits of the left nasal-cranial base lesion.

**Figure 4:**
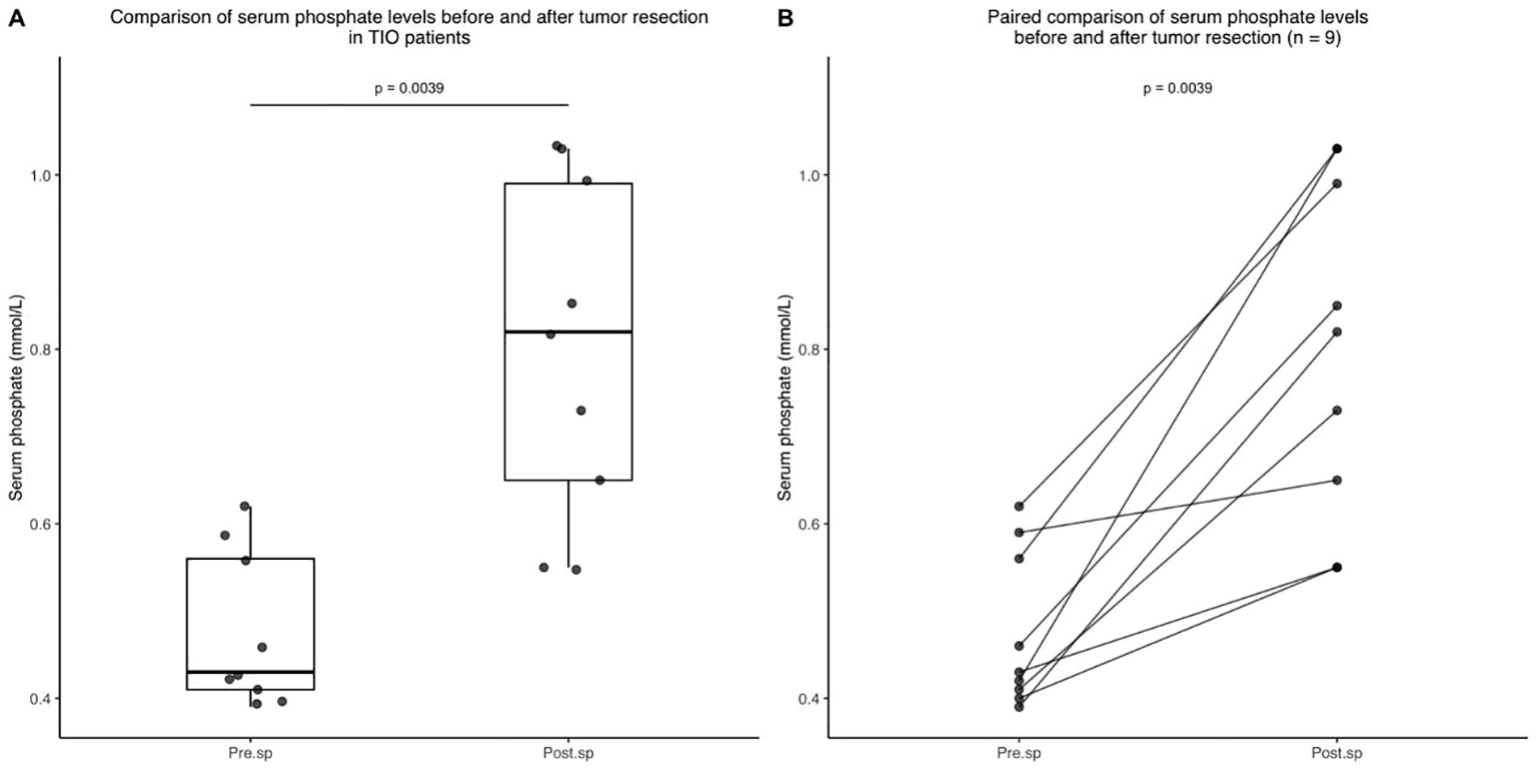
Box plots **(A)** and spaghetti plots **(B)** showing serum phosphate levels in 9 TIO patients before and after tumor resection.

Surgical resection was performed in 9 TIO patients; the remaining 2 patients declined intervention (**Table 4**). Histopathological evaluation of the majority of surgical specimens revealed a phosphaturic mesenchymal tumor (PMT). As exemplified by the eighth case in our cohort, histopathological examination of the left nasal-cranial base lesion confirmed a PMT, composed of ovoid to short spindle-shaped cells, accompanied by focal stromal sclerosis and occasional calcific deposits (**Figure 3**). **Figure 4** illustrates the change in serum phosphorus levels associated with surgery. Tumor excision led to a significant elevation in serum phosphorus (preoperative mean: 0.48 ± 0.09 mmol/L; postoperative mean: 0.80 ± 0.18 mmol/L; p = 0.0039 by paired t-test).

**Table 4:** Changes in serum phosphorus levels and pathological findings in TIO patients.

Patient	Serum phosphorus levels pre-operation	Serum phosphorus levels post-operation	Surgical intervention	Pathological findings	Tumor size
1	0.39	0.82	Yes	Fibrous Histiocytoma	Inaccessible information
2	0.43	0.55	Yes	Phosphaturic Mesenchymal Tumor, PMT	1.8*1.5*0.5cm
3	0.46	0.85	Yes	Radicular Cyst	3.0*2.0*1.0cm
4	0.62	0.99	Yes	Phosphaturic Mesenchymal Tumor, PMT	1.7*1.5*1.2cm
5	0.59	0.65	Yes	Neoplasm of Uncertain Biological Potential	1.0*0.8*0.3cm
6	0.4	0.55	Yes	Consistent with a Neoplastic Process	1.2*2.5*0.8cm
7	0.56	1.03	Yes	Phosphaturic Mesenchymal Tumor, PMT	1.5*1.5*0.6cm
8	0.42	1.03	Yes	Phosphaturic Mesenchymal Tumor, PMT	4.5*3.0*1.2cm
9	0.41	0.73	Yes	Giant Cell Tumor of Bone at the C4 Spinous Process	2.2*1.3cm
10	0.72	–	No	–	–
11	0.71	–	No	–	–

All patients who underwent surgery reported substantial symptomatic relief or complete symptom resolution during follow-up. Notably, two of these patients demonstrated an increase in bone mineral density on postoperative assessment.

## 4 Discussion

Hypophosphatemic osteomalacia is a rare metabolic bone disorder characterized by hypophosphatemia, elevated alkaline phosphatase, and renal phosphate wasting, leading to impaired skeletal mineralization and resultant osteomalacia (or rickets in children). Clinically, it presents with generalized bone pain of obscure etiology and gait disturbance due to muscle weakness. Some patients may experience a noticeable loss of height over several years. The first case was documented by McCance in 1947, yet the causal link between neoplasms and osteomalacia remained elusive until 1959. Subsequent progress in diagnostic imaging and awareness of the disease has led to an increasing number of reported cases in recent decades, though the literature remains dominated by isolated case reports rather than large series (12–15).

The diagnostic workup for TIO begins with a constellation of clinical features, including bone pain, proximal muscle weakness leading to gait abnormalities, functional limitations, and, in some cases, pathological fractures. Minisola et al. reviewed that TIO is frequently misdiagnosed due to overlapping symptoms (bone pain, weakness, fractures) with osteoporosis, causing substantial clinical burden. Pain, skeletal lesions, and muscle weakness due to hypophosphatemia were most reported; patients often suffer impaired mobility or even bedrest, with occasional anxiety, depression, and work loss (16). In our center, the most common clinical features were bone pain, followed by pathological fractures, muscle weakness/fatigue, gait impairment, and height loss. The mean age (± SD) at diagnosis for TIO patients was 51 years (± 14 years), while the mean age at symptom onset was 47 years (± 17 years), with an approximately equal male-to-female ratio (6:5). Consistent with our findings, a study by Abrahamsen et.al (17). indicated that among patients with a suspected diagnosis of TIO, 40% were male and 60% were female. Rendina and colleagues (18) conducted a systematic review and individual patient data analysis of 1725 TIO cases. The diagnosis was made in 843 men (55%) and 689 women (45%).

Key laboratory findings comprise a biochemical profile of mild hypocalcemia, profound hypophosphatemia, elevated alkaline phosphatase, secondary hyperparathyroidism, and characteristically diminished urinary calcium excretion. Notably, urinary phosphate excretion is inappropriately high relative to the degree of hypophosphatemia, with a corresponding marked reduction in both tubular phosphate reabsorption and the renal phosphate threshold (TmP/GFR), confirming a renal phosphate-wasting state. A portion of patients may also exhibit Fanconi syndrome, a feature linked to genetic polymorphisms in XPR1 and SLC34A3 (19). Our analysis highlights serum phosphate, alkaline phosphatase, and TmP/GFR as discriminatory biomarkers for distinguishing TIO from osteoporosis.

Although baseline BMD measurements showed no significant difference between the two groups (**Figure 3**), longitudinal follow-up revealed markedly divergent trajectories. Among two TIO patients evaluated approximately two years after curative tumor resection, lumbar spine BMD increased by 0.229 g/cm² (from 0.873 to 1.102 g/cm²) and 0.366 g/cm²(from 0.847 to 1.213 g/cm²), with corresponding femoral neck BMD gained by 0.116 g/cm²(from 0.595 to 0.711 g/cm²) and 0.392g/cm²(from 0.433 to 0.825 g/cm²), respectively. In contrast, two osteoporosis patients receiving conventional therapy over a comparable interval exhibited only minimal improvements: lumbar spine BMD increased by 0.061 g/cm² (from 0.876 to 0.937 g/cm²) and 0.087 g/cm² (from 0.624 to 0.711 g/cm²), while femoral neck BMD gained by 0.073 g/cm² (from 0.545 to 0.618 g/cm²) and 0.007 g/cm² (from 0.559 to 0.566 g/cm²), respectively. This marked postoperative recovery—substantially outpacing the modest gains observed with anti−osteoporotic agents—is consistent with the findings of Colangelo et al. (20). Collectively, these data suggest that longitudinal BMD assessment offers greater diagnostic insight than a single DXA measurement: a pronounced post-treatment increase in BMD strongly supports a diagnosis of TIO, reflecting rapid mineralization of pre-existing osteoid upon restoration of phosphate homeostasis.

Skeletal X-ray, however, plays a crucial role in the differential diagnosis. Characteristic radiographic findings of osteomalacia include generalized cortical thinning, blurred trabeculation, and vertebral changes such as kyphoscoliosis, biconcave “codfish” deformity, and pseudofractures. In osteoporosis, X-rays typically reveal osteopenic but well-defined trabecular patterns and wedge compression fractures. Additionally, when elevated PTH is present, primary hyperparathyroidism should be considered; it usually presents with hypercalciuria and subperiosteal bone resorption on radiographs. A detailed history regarding hepatitis, drug/toxin/radiation exposure, and familial predisposition is also essential. The absence of metabolic acidosis, glycosuria, and abnormal aminoaciduria helps exclude hereditary causes, renal tubular acidosis, and Fanconi syndrome.

Once the differential diagnosis suggests TIO, a targeted screening protocol is warranted. Physical examination, though imperative, rarely localizes the small tumor directly—only approximately 14% present as a palpable mass. Bosman et al. (21) identified local symptoms in 74 of 895 cases, suggesting that careful history and physical examination may yield localization clues in at least 8.3% of instances, thereby guiding subsequent imaging. A stepwise imaging approach is recommended: initial functional imaging (e.g., somatostatin receptor-based PET/CT), followed by anatomical imaging (CT/MRI) for confirmation. Adjunctive techniques such as selective venous sampling for FGF23 and octreotide scintigraphy may assist in challenging cases. Ultimately, a definitive diagnosis and differentiation from osteoporosis rely on a synthesis of historical clues, clinical signs, distinctive biochemical profiles, skeletal features of X-ray and BMD findings.

Concerning anatomical distribution, a recent 2023 review (3) highlights the extremities (both soft tissue and bone) as the predominant sites for TIO-associated tumors, with craniofacial and sinonasal localizations being less frequent. Our cohort demonstrated a comparable pattern: 36.4% of tumors arose in the head, 45.4% in the lower limbs (including one in the hip), and 18.2% in the trunk. These findings align with the large-scale analysis by Bosman et al. (21), which noted a predilection for the lower limbs (46.4%) and the head/neck region (25.7%), despite the potential for tumors to develop in virtually any anatomical site.

Clinical diagnosis of TIO is challenging and frequently delayed or erroneous. Contributing factors include its nonspecific and variable symptomatology, infrequent assessment of serum phosphate in routine panels, the often occult nature of small causative tumors that evade conventional imaging, and a lack of disease recognition among non-specialists. Consequently, patients commonly undergo evaluations in rheumatology, orthopedics, pain clinics, or general surgery before reaching the correct diagnosis in endocrinology. The reported initial misdiagnosis rate has been reported as 95.1% (22). In our cohort, the median diagnostic delay was 36 months (24–48 months). Every patient had been misdiagnosed at least once, predominantly as osteoporosis (3 cases) or lumbar disc disease (3 cases), followed by hematologic disorders (1 case), hypophosphatemic rickets (1 case), and secondary hyperparathyroidism (1 case); 2 patients remained undiagnosed. To avoid this, enhanced interdisciplinary awareness and multidisciplinary team (MDT) consultations are crucial. We propose a diagnostic cascade: (1) obtain serum phosphate in symptomatic individuals; (2) if hypophosphatemic, measure FGF23 to assess for inappropriately elevated levels; (3) based on these results, initiate molecular imaging for tumor localization.

Osteocyte-derived FGF-23, via Klotho-dependent FGFR1 signaling, suppresses renal phosphate reabsorption and 1,25(OH)_2_D synthesis by downregulating Napi-2a/2c cotransporters (23–27). Most cases of TIO are caused by PMTs that oversecrete FGF-23, and serum FGF-23 levels typically decrease after complete tumor resection. A limitation of the present study is the lack of FGF23 data for the osteoporosis patients. Nonetheless, given that serum phosphate was significantly higher in the osteoporosis group relative to the TIO group, it is reasonable to infer that FGF23 concentrations in the former would also be expected to be lower. However, elevated FGF-23 is not specific to TIO, as comparable increases occur in XLH, ADHR, fibrous dysplasia, post-transplant states, and iron therapy-related hypophosphatemia (28–32), although TIO typically exhibits the highest circulating levels (33). Consequently, an isolated elevation in FGF23 is not diagnostic of TIO. However, FGF-23 may occasionally remain within the normal range. Secreted frizzled-related protein 4 (sFRP4), matrix extracellular phosphoglycoprotein (MEPE), and FGF-7 are other rare non-FGF23 phosphatonins to be considered (34).

Historically, tumors associated with TIO had been categorized as hemangiopericytomas, hemangiomas, giant cell tumors, osteoblastomas, and other entities. The seminal work of Weidner and Santa Cruz (1987) (35), later refined by Folpe and colleagues (2004) (36), established phosphaturic mesenchymal tumor (PMT) as a distinct histopathological entity encompassing the majority of TIO-related neoplasms. Nonetheless, not all tumors responsible for TIO are classified as PMTs. Reported pathologic types associated with TIO include a spectrum beyond PMTs, such as hemangiopericytoma, osteoblastoma, hemangioma, and giant cell tumor. Morphologically, PMTs are heterogeneous neoplasms characterized by a variable admixture of bland spindle cells, osteoid-like matrix, and multinucleated giant cells, typically supported by a prominent, richly vascularized stroma. A systematic review of 895 published cases (21) identified PMT as the dominant histology responsible for TIO (65.4%), with hemangiopericytoma and giant cell tumor being the next most frequent. Restricting the analysis to cases reported after the establishment of the PMT diagnostic criteria by Folpe et al. in 2004 (36), the PMT prevalence rose to 75.5%. Within that review, histologic assessment indicated malignancy in 56 of 579 tumors (9.7%). Similarly, in our study, tumors with suspected malignant features accounted for 9% (1/11) of cases, aligning closely with this reported rate.

Complete surgical excision stands as the primary and potentially definitive intervention for TIO. The resection of PMTs in soft tissue locations is often complicated by their infiltrative growth pattern into adjacent tissues. Similarly, in bone, these tumors may exhibit permeation through cartilage gaps and generate copious osteoid-like matrix, imparting a histologic resemblance to osteosarcoma (1). Consequently, to minimize the risk of persistence or recurrent disease, wide local excision (as opposed to intralesional curettage) is recommended upon accurate tumor localization. A rapid normalization of serum phosphate, often occurring within hours to days after surgery, coupled with substantial symptomatic relief (e.g., reduction in bone pain and weakness) over subsequent weeks to months, constitutes the therapeutic and diagnostic hallmark of TIO. In cases where surgical cure is not achievable—due to occult tumor location, surgical ineligibility, or postoperative relapse—medical therapy becomes the mainstay. The foundational regimen consists of co-administration of oral phosphate supplements and active vitamin D (calcitriol). The roles of cinacalcet and somatostatin analogs remain less defined, supported by limited evidence and inconsistent clinical outcomes. In Patient 3 of our study, both 18F-FDG PET/CT and 68Ga-DOTATATE PET/CT demonstrated focal radiotracer avidity in the left lateral femur, initially suggestive of a neuroendocrine neoplasm. Subsequent surgical pathology identified the mass as a phosphaturic mesenchymal tumor (PMT) in the para-femoral soft tissue. Based on the principle that PMTs frequently express somatostatin receptors (SSTRs)—explaining the positive SSTR imaging—the patient was treated with subcutaneous octreotide acetate (0.1 mg q12h). This intervention failed to elicit a biochemical (normalization of serum phosphate) or clinical response. The therapeutic failure may be attributed to two factors: (1) The chosen short-acting octreotide formulation, used at a low dose given the patient’s history of cholelithiasis, may have been subtherapeutic due to its pharmacokinetic profile; a broader-spectrum somatostatin analog like pasireotide might offer theoretical advantages. (2) The discordance between the pharmacologic profile of octreotide (non-selective for SSTR2 vs. SSTR5) and the imaging characteristics of 68Ga-DOTATATE (highly selective for SSTR2) raises the possibility that the tumor’s phenotype was dominated by SSTR2 expression (37), a premise that warrants validation via receptor-specific immunohistochemistry. Recent years have witnessed active research into FGFR1-targeted agents. The human monoclonal FGF23 antibody, Burosumab (KRN23), has shown promising efficacy and a favorable safety profile in managing both XLH and non-resectable TIO. Furthermore, emerging therapeutic avenues such as pan-FGF receptor inhibitors and peptide receptor radionuclide therapy (PRRT) hold potential as future alternatives for patients ineligible for definitive surgery.

Our findings identified serum phosphate, alkaline phosphatase, and TmP/GFR as useful discriminators between condition TIO and POP. For symptomatic patients (e.g., with bone pain or muscle weakness), we recommend initial evaluation with these biochemical parameters. If hypophosphatemia is confirmed, FGF23 measurement is warranted after excluding other known causes of elevated FGF23. Findings from X-ray and BMD can then guide the decision to pursue tumor localization via functional molecular imaging. The diagnostic gold standard remains: (1) histopathological confirmation of a responsible tumor, (2) rapid normalization of serum phosphate levels postoperatively, and (3) subsequent significant clinical improvement.

### Study limitations

The inherent rarity of TIO constrains our analysis to a relatively small, single-center cohort accrued over twenty years. Case ascertainment may be incomplete, as patients could have been evaluated and treated in other clinical units. Diagnostic challenges are compounded by variable recognition of TIO among both treating clinicians and pathologists, leading to a lack of definitive PMT diagnoses in some historical cases. Moreover, advanced diagnostic modalities—specifically somatostatin receptor imaging (e.g., 68Ga-DOTATATE PET/CT) and serum FGF23 measurement—have only been integrated into our diagnostic pathway within the last five years and are not yet widely utilized across all relevant departments, especially the lack of available FGF23 data for the osteoporosis control group, further contributing to potential selection bias.

In conclusion, by detailing our institutional experience with TIO, we aim to elevate disease recognition among a broader range of specialists, foster regional multidisciplinary collaboration, advocate for the integration of molecular imaging into diagnostic algorithms, and thereby reduce diagnostic and therapeutic delays often faced by patients. Our findings highlight both the importance and the limitations of FGF23 testing, while underscoring the critical value of bone metabolism assessment and long-term follow-up.

## Data Availability

The original contributions presented in the study are included in the article/supplementary material. Further inquiries can be directed to the corresponding author.

## References

[1] Folpe AL . Phosphaturic mesenchymal tumors: a review and update. Semin Diagn Pathol. (2019) 36(4):260–8. doi: 10.1053/j.semdp.2019.07.002 31301876

[2] White KE Jonsson KB Carn G Hampson G Spector TD Mannstadt M . The autosomal dominant hypophosphatemic rickets (ADHR) gene is a secreted polypeptide overexpressed by tumors that cause phosphate wasting. J Clin Endocrinol Metab. (2001) 86(2):497–500. doi: 10.1210/jc.86.2.497 11157998

[3] Minisola S Fukumoto S Xia W Corsi A Colangelo L Scillitani A . Tumor-induced osteomalacia: a comprehensive review. Endocr Rev. (2023) 44(2):323–53. doi: 10.1210/endrev/bnac026 36327295

[4] Mazzaferro S Pasquali M Pirro G Rotondi S Tartaglione L . The bone and the kidney. Arch Biochem Biophys. (2010) 503(1):95–102. doi: 10.1016/j.abb.2010.06.028 20599669

[5] Haffner D Emma F Eastwood DM Biosse Duplan M Bacchetta J Schnabel D . Clinical practice recommendations for the diagnosis and management of X-linked hypophosphataemia. Nat Rev Nephrol. (2019) 15(7):435–55. doi: 10.1038/s41581-019-0152-5 31068690 PMC7136170

[6] Brandi ML Clunie GPR Houillier P Jan de Beur SM Minisola S Oheim R . Challenges in the management of tumor-induced osteomalacia (TIO). Bone. (2021) 152:116064. doi: 10.1016/j.bone.2021.116064 34147708

[7] Ng E Ashkar C Seeman E Schneider HG Nguyen H Ebeling PR . A low serum alkaline phosphatase may signal hypophosphatasia in osteoporosis clinic patients. Osteoporos Int. (2023) 34(2):327–37. doi: 10.1007/s00198-022-06597-3 36434431

[8] Jha S Chapman M Roszko K . When low bone mineral density and fractures is not osteoporosis. Curr Osteoporos Rep. (2019) 17(5):324–32. doi: 10.1007/s11914-019-00529-7 31468499 PMC6819255

[9] Walton RJ Bijvoet OL . Nomogram for derivation of renal threshold phosphate concentration. Lancet. (1975) 2(7929):309–10. doi: 10.1016/s0140-6736(75)92736-1 50513

[10] Souberbielle JC Prie D Piketty ML Rothenbuhler A Delanaye P Chanson P . Evaluation of a new fully automated assay for plasma intact FGF23. Calcif Tissue Int. (2017) 101(5):510–8. doi: 10.1007/s00223-017-0307-y 28761972

[11] WHO Classification of Tumours Editorial Board . Soft Tissue and Bone Tumours. Lyon: International Agency for Research on Cancer (2020). 3

[12] Annamalai AK Shinto A Aram S Prabhu VA Santhi R Gopalakrishnan C . Tumor-induced osteomalacia. Kidney Int. (2022) 102(5):1194. doi: 10.1016/j.kint.2022.04.042 36272746

[13] Thi HN Manh CP Tuan LT Le Thi LA Thanh NN Vilaiyuk S . Tumor-induced osteomalacia in a boy with maxillary ossifying fibroma. J Clin Res Pediatr Endocrinol. (2023) 15(4):421–5. doi: 10.4274/jcrpe.galenos.2022.2021-8-14 35135186 PMC10683546

[14] Zhang Z Li J Zhang Z Shao Z . Tumor-induced osteomalacia: a case report and etiological analysis with literature review. Orthop Surg. (2023) 15(12):3342–52. doi: 10.1111/os.13901 37933469 PMC10694022

[15] Colangelo L Sonato C Cipriani C Pepe J Farinacci G Palmisano B . Occipital bone and tumor-induced osteomalacia: a rare tumor site for an uncommon paraneoplastic syndrome. Arch Osteoporos. (2023) 18(1):94. doi: 10.1007/s11657-023-01305-y 37436671 PMC10338621

[16] Minisola S Barlassina A Vincent SA Wood S Williams A . A literature review to understand the burden of disease in people living with tumour-induced osteomalacia. Osteoporos Int. (2022) 33(9):1845–57. doi: 10.1007/s00198-022-06432-9 35643939 PMC9463218

[17] Abrahamsen B Smith CD Minisola S . Epidemiology of tumor-induced osteomalacia in Denmark. Calcif Tissue Int. (2021) 109(2):147–56. doi: 10.1007/s00223-021-00843-2 33818653 PMC8273058

[18] Rendina D Abate V Cacace G D'Elia L De Filippo G Del Vecchio S . Tumor-induced osteomalacia: a systematic review and individual patient's data analysis. J Clin Endocrinol Metab. (2022) 107(8):e3428-e36. doi: 10.1210/clinem/dgac253 35468192

[19] Jiang Y Li X Feng J Li M Wang O Xing XP . The genetic polymorphisms of XPR1 and SCL34A3 are associated with Fanconi syndrome in Chinese patients of tumor-induced osteomalacia. J Endocrinol Invest. (2021) 44(4):773–80. doi: 10.1007/s40618-020-01371-w 32725396

[20] Colangelo L Pepe J Nieddu L Sonato C Scillitani A Diacinti D . Long-term bone mineral density changes after surgical cure of patients with tumor-induced osteomalacia. Osteoporos Int. (2020) 31(7):1383–7. doi: 10.1007/s00198-020-05369-1 32185436

[21] Bosman A Palermo A Vanderhulst J De Beur SMJ Fukumoto S Minisola S . Tumor-induced osteomalacia: a systematic clinical review of 895 cases. Calcif Tissue Int. (2022) 111(4):367–79. doi: 10.1007/s00223-022-01005-8 35857061 PMC9474374

[22] Feng J Jiang Y Wang O Li M Xing X Huo L . The diagnostic dilemma of tumor induced osteomalacia: a retrospective analysis of 144 cases. Endocr J. (2017) 64(7):675–83. doi: 10.1507/endocrj.ej16-0587 28450684

[23] Kurosu H Kuro OM . The Klotho gene family as a regulator of endocrine fibroblast growth factors. Mol Cell Endocrinol. (2009) 299(1):72–8. doi: 10.1016/j.mce.2008.10.052 19063940

[24] Richter B Faul C . FGF23 actions on target tissues-with and without Klotho. Front Endocrinol (Lausanne). (2018) 9:189. doi: 10.3389/fendo.2018.00189 29770125 PMC5940753

[25] Minisola S Peacock M Fukumoto S Cipriani C Pepe J Tella SH . Tumour-induced osteomalacia. Nat Rev Dis Primers. (2017) 3:17044. doi: 10.1038/nrdp.2017.44 28703220

[26] Murali SK Roschger P Zeitz U Klaushofer K Andrukhova O Erben RG . FGF23 regulates bone mineralization in a 1,25(OH)2 D3 and Klotho-independent manner. J Bone Miner Res. (2016) 31(1):129–42. doi: 10.1002/jbmr.2606 26235988

[27] Latic N Erben RG . FGF23 and vitamin D metabolism. JBMR Plus. (2021) 5(12):e10558. doi: 10.1002/jbm4.10558 34950827 PMC8674776

[28] Shimada T Muto T Urakawa I Yoneya T Yamazaki Y Okawa K . Mutant FGF-23 responsible for autosomal dominant hypophosphatemic rickets is resistant to proteolytic cleavage and causes hypophosphatemia in vivo. Endocrinology. (2002) 143(8):3179–82. doi: 10.1210/en.143.8.3179 12130585

[29] Takashi Y Kawanami D Fukumoto S . FGF23 and hypophosphatemic rickets/osteomalacia. Curr Osteoporos Rep. (2021) 19(6):669–75. doi: 10.1007/s11914-021-00709-4 34755323

[30] Bhattacharyya N Wiench M Dumitrescu C Connolly BM Bugge TH Patel HV . Mechanism of FGF23 processing in fibrous dysplasia. J Bone Miner Res. (2012) 27(5):1132–41. doi: 10.1002/jbmr.1546 22247037 PMC7448291

[31] Bhan I Shah A Holmes J Isakova T Gutierrez O Burnett SM . Post-transplant hypophosphatemia: tertiary 'hyper-phosphatoninism'?. Kidney Int. (2006) 70(8):1486–94. doi: 10.1038/sj.ki.5001788 16941023

[32] Ichchou L Rist S Lespessailles E Prie D Benhamou CL . Reversible increase in FGF23 in a hypophosphatemic renal and bone disease linked to antiviral therapy by adefovir. Joint Bone Spine. (2013) 80(6):668–9. doi: 10.1016/j.jbspin.2013.02.014 23583281

[33] Endo I Fukumoto S Ozono K Namba N Tanaka H Inoue D . Clinical usefulness of measurement of fibroblast growth factor 23 (FGF23) in hypophosphatemic patients: proposal of diagnostic criteria using FGF23 measurement. Bone. (2008) 42(6):1235–9. doi: 10.1016/j.bone.2008.02.014 18396126

[34] Montanari A Pirini MG Lotrecchiano L Di Prinzio L Zavatta G . Phosphaturic mesenchymal tumors with or without phosphate metabolism derangements. Curr Oncol. (2023) 30(8):7478–88. doi: 10.3390/curroncol30080541 37623022 PMC10453447

[35] Weidner N Santa Cruz D . Phosphaturic mesenchymal tumors: a polymorphous group causing osteomalacia or rickets. Cancer. (1987) 59(8):1442–54. doi: 10.1002/1097-0142(19870415)59:8<1442::aid-cncr2820590810>3.0.co;2-q 3545439

[36] Folpe AL Fanburg-Smith JC Billings SD Bisceglia M Bertoni F Cho JY . Most osteomalacia-associated mesenchymal tumors are a single histopathologic entity: an analysis of 32 cases and a comprehensive review of the literature. Am J Surg Pathol. (2004) 28(1):1–30. doi: 10.1097/00000478-200401000-00001 14707860

[37] Rayamajhi SJ Yeh R Wong T Dumeer S Mittal BR Remotti F . Tumor-induced osteomalacia - current imaging modalities and a systematic approach for tumor localization. Clin Imaging. (2019) 56:114–23. doi: 10.1016/j.clinimag.2019.04.007 31029010

